# A multi-cellular 3D bioprinting approach for vascularized heart tissue engineering based on HUVECs and iPSC-derived cardiomyocytes

**DOI:** 10.1038/s41598-018-31848-x

**Published:** 2018-09-10

**Authors:** Fabio Maiullari, Marco Costantini, Marika Milan, Valentina Pace, Maila Chirivì, Silvia Maiullari, Alberto Rainer, Denisa Baci, Hany El-Sayed Marei, Dror Seliktar, Cesare Gargioli, Claudia Bearzi, Roberto Rizzi

**Affiliations:** 1 Operational Research Unit, Fondazione di Ricerca e Cura Giovanni Paolo II, Largo Gemelli, Campobasso, 86100 Italy; 2 Institute of Cell Biology and Neurobiology (IBCN), National Research Council of Italy (CNR), Monterotondo Scalo, Rome, 00015 Italy; 3 Institute of Physical Chemistry, Polish Academy of Sciences, Warsaw, 01224 Poland; 4 Tissue Engineering Lab, Università Campus Bio-Medico di Roma, Rome, 00128 Italy; 5 IRCCS MultiMedica, Scientific and Technology Pole, Milan, 20100 Italy; 6 Department of Cytology and Histology, Faculty of Veterinary Medicine, Mansoura University, Mansoura, 35116 Egypt; 7 Department of Biomedical Engineering, Technion Institute, Haifa, 32000 Israel; 8 Department of Biology, Tor Vergata Rome University, Rome, 00133 Italy

## Abstract

The myocardium behaves like a sophisticated orchestra that expresses its true potential only if each member performs the correct task harmonically. Recapitulating its complexity within engineered 3D functional constructs with tailored biological and mechanical properties, is one of the current scientific priorities in the field of regenerative medicine and tissue engineering. In this study, driven by the necessity of fabricating advanced model of cardiac tissue, we present an innovative approach consisting of heterogeneous, multi-cellular constructs composed of Human Umbilical Vein Endothelial Cells (HUVECs) and induced pluripotent cell-derived cardiomyocytes (iPSC-CMs). Cells were encapsulated within hydrogel strands containing alginate and PEG-Fibrinogen (PF) and extruded through a custom microfluidic printing head (MPH) that allows to precisely tailor their 3D spatial deposition, guaranteeing a high printing fidelity and resolution. We obtained a 3D cardiac tissue compose of iPSC-derived CMs with a high orientation index imposed by the different defined geometries and blood vessel-like shapes generated by HUVECs which, as demonstrated by *in vivo* grafting, better support the integration of the engineered cardiac tissue with host’s vasculature.

## Introduction

According to the last report of the World Health Organization (WHO), cardiovascular diseases (CVDs), such as genetic or ischemic heart disease, are still the leading cause of mortality in the industrialized world^1^, with a rate of 23 million new patients diagnosed worldwide every year^2^. Such diseases affect the functions of the myocardium causing irreversible damages to the tissue that generally leads to heart failure, a condition characterized by a decrease in contractile capacity below a critical threshold^3^.

Currently, despite the constant efforts of the researchers to improve treatments for cardiac insults, there is no effective cure for heart failure, with the exception of heart transplantation, which, due to the extremely invasive nature of the surgery and the shortage of organ donors, is applicable only for a limited cohort of patients. Furthermore, complications of state-of-the-art immunotherapeutic drugs and high risk of rejection restrict the possibility of recovery.

The pivotal problem is that cardiac muscular cells in humans and other mammals show a very limited capacity for self-renewal in response to injury, which is in contrast to the more widespread regenerative capacity in lower vertebrates, such as zebrafish^4^.

So far, bone marrow (BM-MSC) or adipose tissue (ASC) derived-mesenchymal cells^5^, Skeletal Myoblasts (SKM)^6,7^, Embryonic Stem Cells (ESC)^8^ and resident Cardiac Stem Cells (CSC)^9,10^ have been tested to treat myocardium injuries. However, the results obtained are not univocal and are often limited to neo-angiogenesis due to paracrine activity of transplanted cells or to a limited functional integration of immature cardiomyocytes (CM)^11^.

These medical challenges have raised the need for innovative and more effective cell-based approaches that are currently the subject of numerous research studies^12,13^. To this aim, the tissue engineering and regenerative medicine approaches revealed great potential as alternative options, creating constructs for repairing or replacing macroscopic part of cardiovascular tissue^14–17^. Moreover, modern technologies for the transplantation of human organs - with their countless challenges and high costs - are ripe for making a revolution to innovation and process optimization.

Nowadays, one of the most advanced technologies used to fabricate engineered tissues is based on additive manufacturing systems: this technique represent a fast and cost effective biofabrication approaches, able to create macroscopic 3D objects with high precision, high resolution and high repeatability^18–20^. In particular, 3D bioprinting has gaining attention in the research community because it allows the simultaneous deposition of cells and biomaterials in a *layer-by-layer* fashion, to form 3D well-organized heterogeneous structures able to morphologically and structurally recapitulate the complex biological tissue architectures. Hence, 3D bioprinting could have the capacity to support and develop the true therapeutic potential of stem cells, which play an increasingly pivotal role in regenerative medicine.

Here, we present a multidisciplinary approach that integrates the use of 3D bioprinting in combination with induced pluripotent stem cell-derived CM (iPSC-CM) and HUVEC aiming at fabricating both an *in vitro* and *in vivo* faithful model of vascularized cardiac tissue. Specifically, iPSCs seem to be the best candidate for cardiac tissue engineering for several reasons. These cells, can be derived from an adult patients’ own cells harvested from non-invasive skin biopsies, they possess unlimited proliferation capacity, and they can be differentiated into any type of tissue. Such outstanding pluripotency is comparable to the one exhibited by embryonic stem cells (ESC) with the real advantage of overcoming ethical concerns. Furthermore, it is well established that non-muscular heart cells, such as fibroblasts and vascular cells, play a key role in myocardial function^21,22^ emphasizing the need for multi-cellular patches in cardiac regenerative medicine. Hence, in this study, we first show a well organized and homogeneous 3D model of murine iPSC cultures which maintain the stemness after the bioprinting process and without the use of inactivated embryonic fibroblast feeder layers. Then, we demonstrate that our microfluidic printing head (MPH) guarantees high-resolution bioprinting generating heterogeneous constructs composed of iPSC-derived CM and HUVEC with different spatial distribution, enabling the fabrication of 3D cardiac tissue models enriched with a vascular network. Finally, we determine that such constructs after *in vivo* implantation i) amplify the cell differentiation potential toward the cardiac phenotype, ii) improve the overall alignment of CM (a key property for generating a synchronous and performing cardiac contraction) and iii) favour the formation of large vessels.

## Results

### Setting-up MPH-bioprinting process

Recapitulating the complexity and heterogeneity of the cardiac tissue *in vitro* is a fundamental requirement to develop reliable and functional models of this organ. To address this great biotechnological challenge, we developed a sophisticated microfluidic-based printing head (MPH) for the simultaneous extrusion of multiple bioinks.

**Figure 1:**
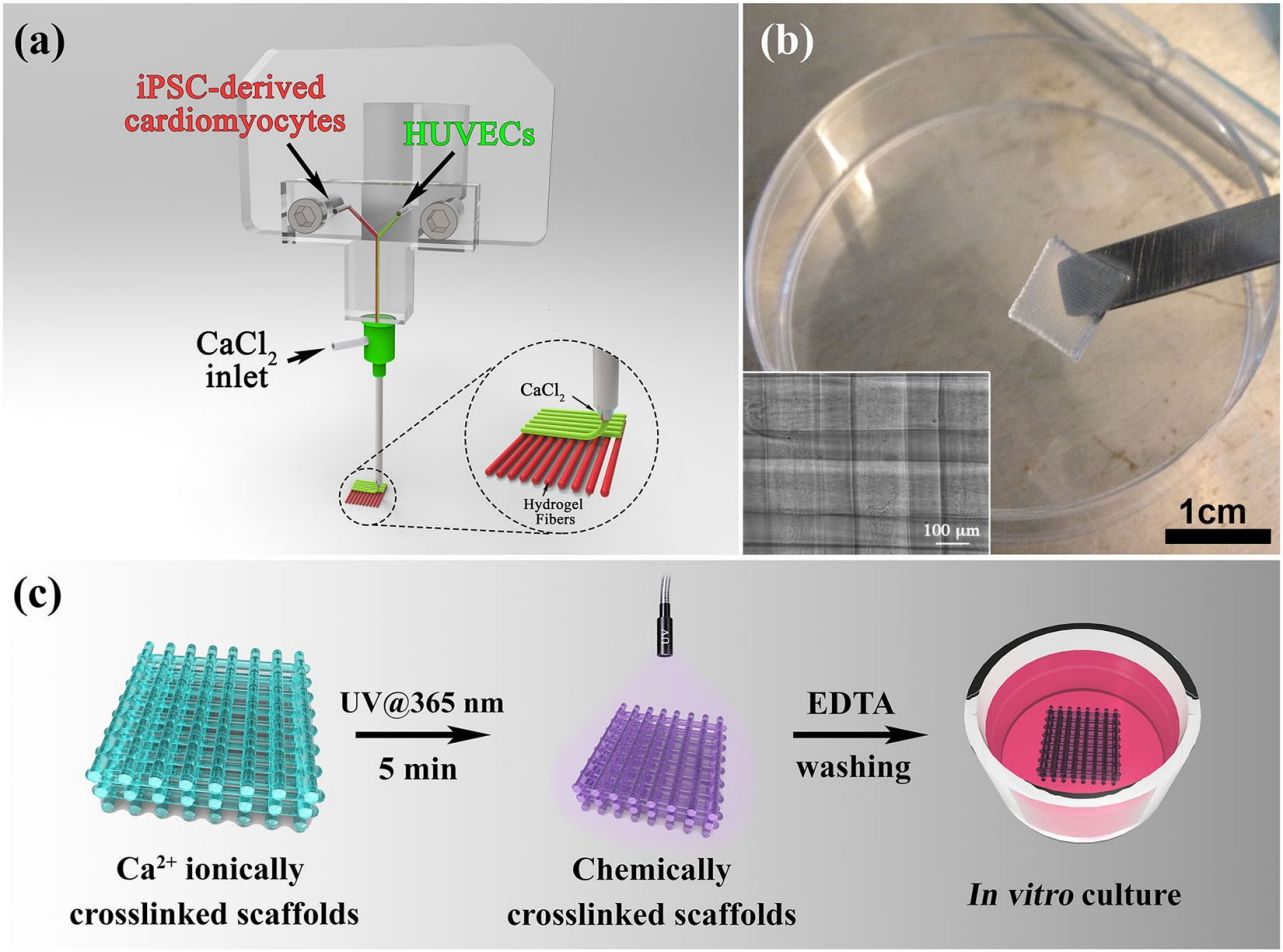
3D bioprinting. (**a**) Schematics representation of the microfluidic printing head (MPH) coupled to a co-axial nozzle extruder employed to simultaneously bioprint iPSC-derived cardiomyocytes and HUVEC cells. (**b**) Bioprinted cellularized 10-layers thick construct. Higher magnification image of the same construct is presented in the box below. Scale bars represent 1 cm and 100 µm, respectively (**c**) Diagram showing the procedure for the fabrication of covalently crosslinked PEG-Fibrinogen based scaffolds prior to undergo *in vitro* culture.

The MPH was designed in a way that the outlet channel is fluidically connected to a co-axial needle system that serves as the extruder for the immediate gelification of the laid hydrogel fibers (Fig. 1a)^23^. To exploit this feature, in this study we formulated bioinks containing alginate (ALG) and a semi-synthetic biopolymer, namely PEG monoacrylate-fibrinogen (PF). These two biopolymers have different roles in the bioprinting process: in fact, alginate serves only as a temporary material template allowing controlled deposition of hydrogel fibers through the custom coaxial extruder, while PF provides a tailored matrix (stabilized by UV crosslinking) for cell spreading and differentiation during long *in vitro* cultures.

Thanks to the simultaneous co-extrusion of the bioink and CaCl_2_ solution, hydrogel fibers were formed immediately at the tip of the inner needle and precisely deposited to assemble high-resolution scaffolds (Fig. 1b). To further stabilize the printed structures, samples underwent a secondary UV-crosslinking step in which the vinyl moieties of PF were polymerized for 5 min (λ = 365 nm, UV dose = 1.3 mW/cm^2^) to form a covalently cross-linked network. Finally, prior to start *in vitro* culturing, the large amount of ALG was removed from the 3D printed structures by washing the samples with an EDTA solution (a strong chelating agent). This is a crucial step since alginate does not represent an ideal matrix for culturing iPSC due to its bioinertness (i.e. lack of adhesion moieties) and *in vivo* limited biodegradation. In Fig. 1c, a brief schematic of sample post-printing treatment is reported.

### iPSC characterization and bioprinting

iPSCs were generated from neonatal mouse fibroblasts (1–3 days) through bicistronic vectors viral transduction carrying the oct4-sox2 and oct4-kfl4 gene cocktail (for the details, see materials and methods section). The pluripotency of the iPSC lines was verified by qRT-PCR, which demonstrated a similar expression of oct4, sox2 and nanog -stem genes- between the derived lines and embryonic stem cells. These results were further confirmed by immunofluorescence for stemness markers (SSEA1, Oct4, Nanog – see supplementary Figure S2). Such preliminary experiments were fundamental as there is a very limited literature regarding iPSC bioprinting and potential negative factors related to this technique might be still unknown^24,25^.

Hence, we firstly evaluated the interference of the bioprinting process on the proliferation and stemness of iPSC, benchmarking the obtained results against those obtained encapsulating iPSC in a bulk hydrogel. Undifferentiated iPSC were resuspended in the bioink (containing 4% w/w ALG and 1% w/w PF) at a concentration of 8 × 10^6^ cells/mL, bioprinted and cultured for 14 days in propagation medium composed of KnockOut DMEM (Invitrogen) containing 20% KnockOut serum. In parallel, bulk cylindrical hydrogel with the same chemical composition and iPSC density were prepared by casting the bioink in silicon moulds.

**Figure 2:**
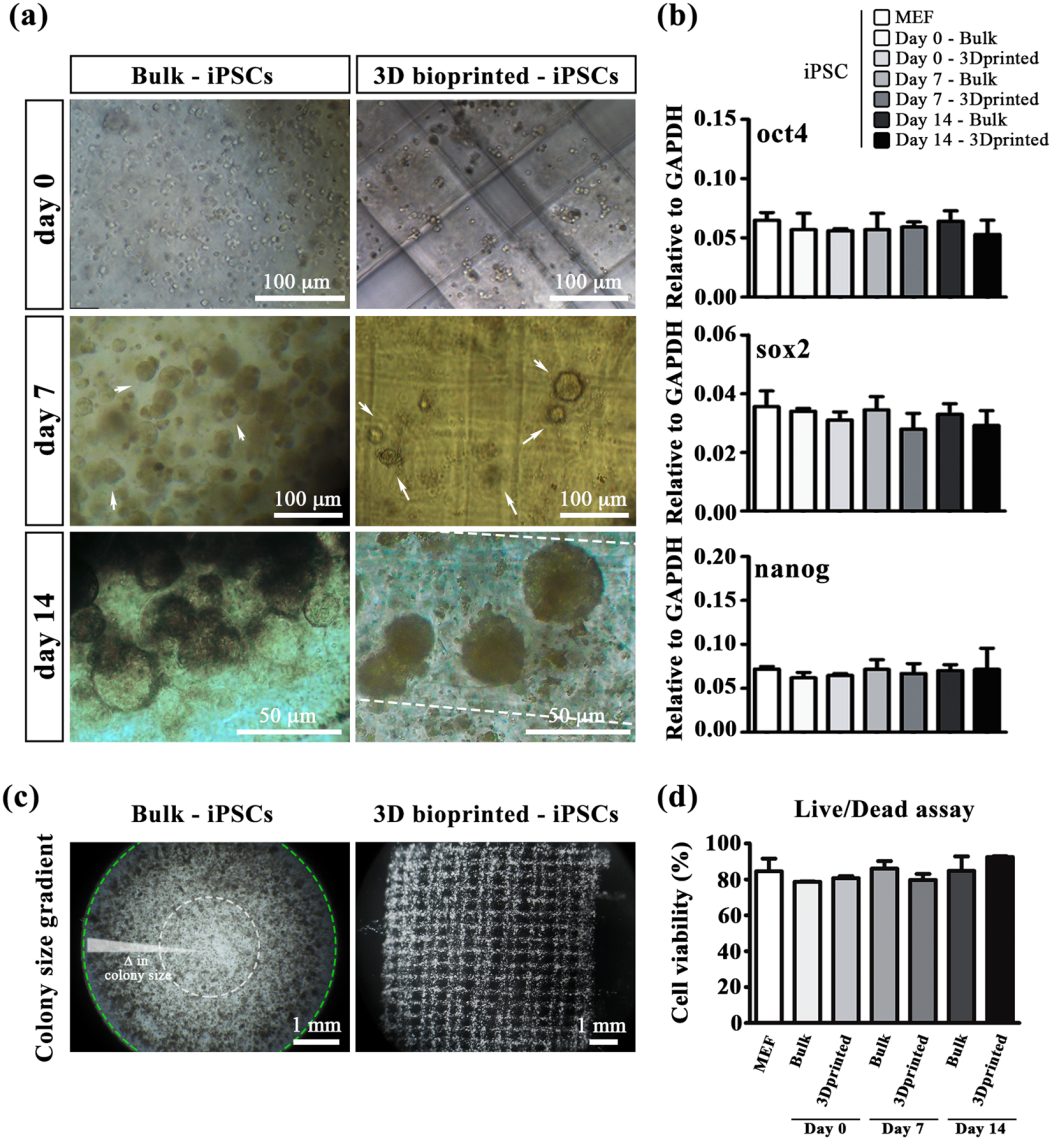
iPSC cultured in 3D scaffolds. (**a**) Representative images of iPSC morphology grown in bulk and iPSC 3D printed at day 0, 7 and 14. Scale bars represent 50 μm and 100 μm, respectively. (**b**) QRT-PCR analysis for stemness genes, such as oct4, sox2 and nanog, in iPSC cultured on MEF feeder layer, bulk hydrogel and 3D bioprinted scaffold at day 0, 7 and 14. Error bars represent mean ± SEM. Student’s t test did not evidence significant differences in gene expression among the different tested conditions. N = 4. **(c)** Colony size gradient of iPSC cultured in bulk and 3D bioprinted at day 14. Scale bars represent 1 mm. (**d**) Live/dead assay graph relative to bulk and 3D bioprinted-iPSCs after 14 days of culture.

As shown in Fig. 2, iPSC soon after encapsulation in bulk hydrogels or bioprinting appeared as single cells homogeneously distributed throughout the whole volume of the samples. However, over culturing time, single iPSC started creating monoclonal colonies that appear as cell agglomerates (Fig. 2a - day 7, white arrows). This is a common feature of proliferating iPSC that can be observed also during their culture on MEF feeder layer^26^. Interestingly, after 14 days of culture, we noticed a significant heterogeneity in the size of colonies in the bulk hydrogels (Fig. 2a), with a negative gradient in size from the most external to central part of the samples (i.e. from the green to the white circle – Fig. 2c). This effect can be easily explained because of diffusion of oxygen and nutrients within the bulk hydrogels that reasonably are more available close to the surface of the sample and less available in the most internal volume. On the contrary, bioprinted sample did not evidence any heterogeneity in the size of colonies thanks to their porous structure that guaranteed an accurate supply of nutrients in all areas of the constructs. Live/dead assay relative to bulk and 3D bioprinted-iPSCs after 14 days of culture showed that the cell viability is assessed between 80 and 90% in all conditions (Fig. 2d). Representative images of live/dead assay was reported in Supplementary Figure S3.

**Figure 3:**
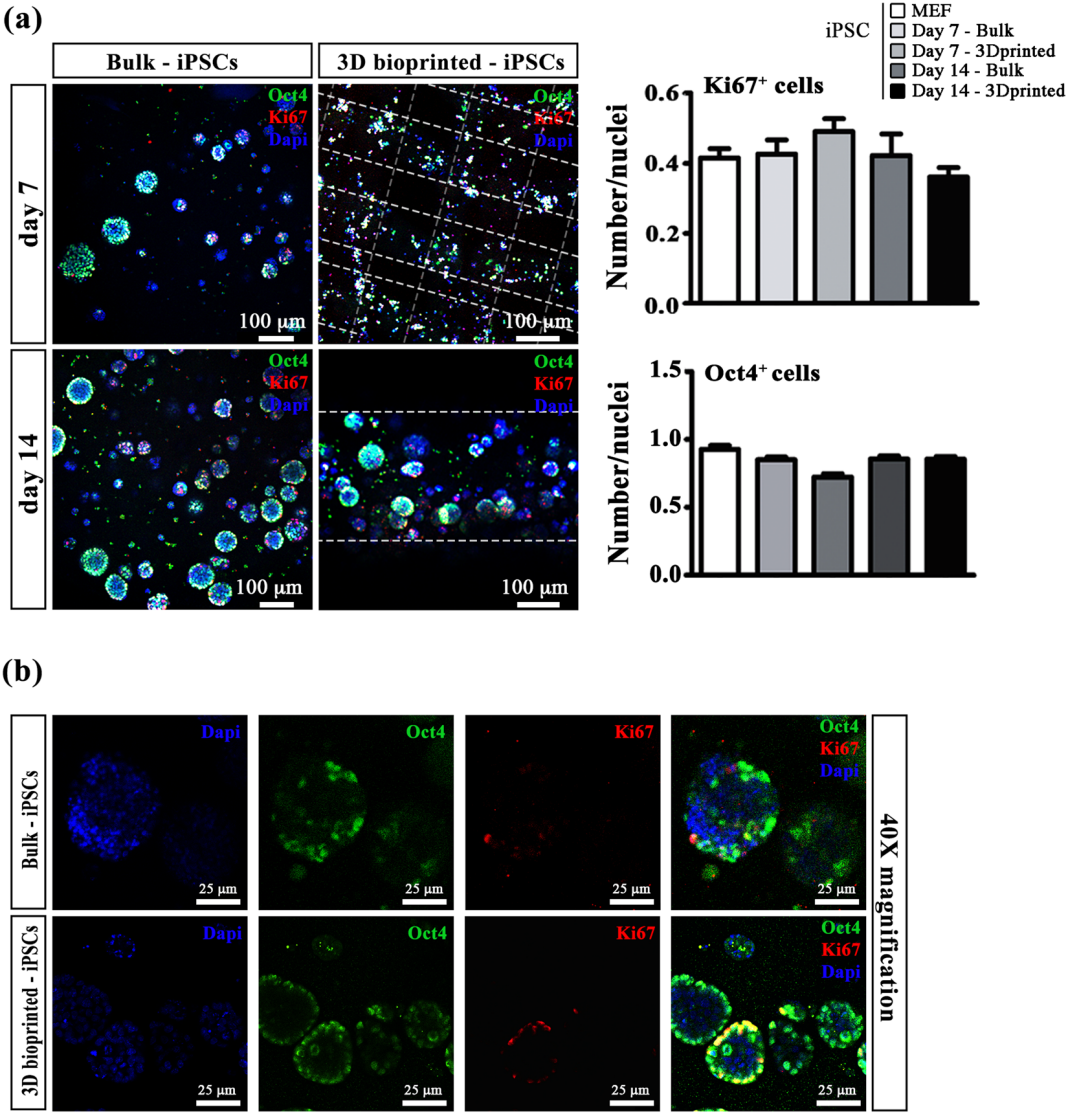
Immunofluorescence assay in iPSC cultured in 3D scaffolds. (**a**) Immunofluorescence for Oct4 (green) and Ki67 (red) in iPSC grown in Bulk and in 3D printed scaffold at day 7 and 14. Scale bars represent 100 μm (left panel) The graphs indicate the fraction of cells positive for Ki67 and Oct4 at day 7 and 1(right panel). N = 4. (**b**) Single channel and merged images of Oct4 and Ki67 positive cells grown in Bulk and in 3D bioprinted constructs at day 14. Magnification: 40X. Scale bars represent 25 μm.

To evaluate quantitatively potential differences induced by bioprinting process in the stemness and proliferation of iPSC, the expression of selected stemness genes was analysed for both scaffolding conditions by qRT-PCR. Notably, the expression levels of oct4, sox2 and nanog genes obtained in both 3D conditions and for all the time points were comparable with those grown on MEF (used as day 0 2D control – see Fig. 2b). The maintenance of stem cell markers was further confirmed by immunofluorescence assay revealing Oct4 expression in all conditions (Fig. 3a–b).

A similar proliferation rate up to 14 days among the two types of 3D scaffolds and the standard 2D culture condition (i.e. iPSC cultured on MEF) was demonstrated by immunofluorescence for Ki67 (proliferation marker) attesting that the bioprinting process did not affect any fundamental iPSC properties (Fig. 3).

### 3D Bioprinting of iPSC-derived CM

In the second part of this study, iPSCs were subjected to a differentiation protocol towards the cardiac phenotype. To this end, murine iPSCs were stimulated with BMP2 for 12 h and cultured in suspended conditions to promote the formation of EB^27^ (schematic differentiation protocol is shown in supplementary Figure S4). Cells were cultured on gelatin-coated dishes until large areas of self-contraction appeared (supplementary video 1) demonstrating the organization of contractile machinery and attesting the cardiac commitment.

Similarly, to undifferentiated iPSCs, iPSC-derived CMs were used to fabricate two type of 3D scaffolds – bulk and 3D bioprinted hydrogels – that were cultured *in vitro* up to 2 weeks. Since CMs need to reach high confluence with the formation of tight intracellular junctions to function properly, in these experiments we employed a higher initial cell concentration of 40 × 10^6^ cells/mL. Moreover, accordingly to our previous study^28^, the stiffness of the final hydrogels was slightly increased with the addition of 1% PEG-DA monomer to better support CM functionality.

**Figure 4:**
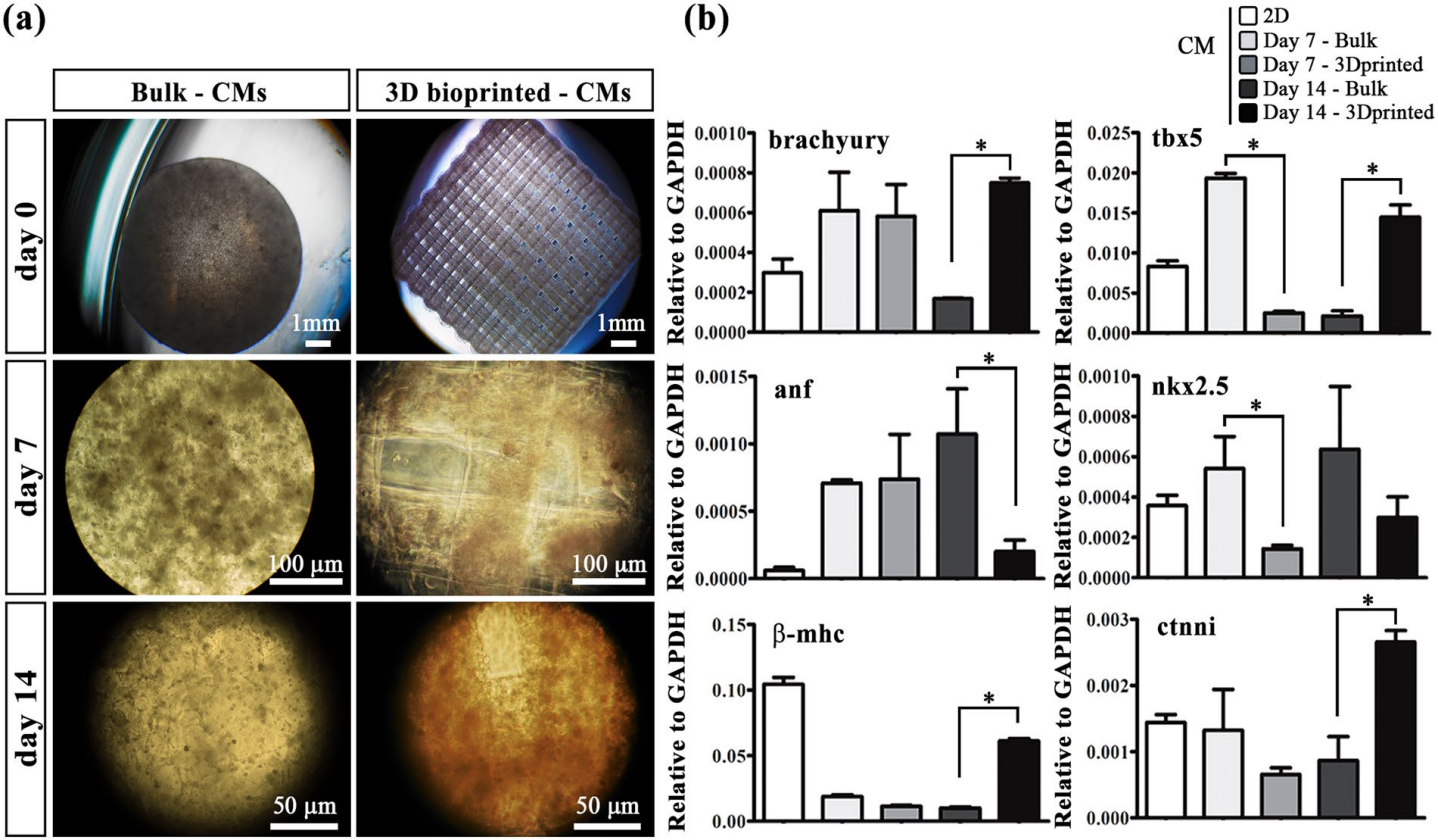
iPSC-derived CM cultured in bulk or 3Dprinted scaffolds. (**a**) CM morphology at day 0, 7 and 14. Scale bars represent: upper panel 1 mm, middle panel 100 μm, lower panel 50 μm. (**b**) Relative gene expression related to cardiac early genes (brachyury, tbx5, anf, nkx2.5, β-mhc) and cardiac late gene (ctnni) in 3D printed CM compared to CM in bulk structures and 2D cultures at day 7 and 14. Error bars represent ± SEM. Student’s t test, *p < 0.05; N = 5.

At selected time points (0, 7 and 14 days), quantitatively (qRT-PCR) and qualitatively (immunofluorescence) assays were used to evaluate the differentiation status of iPSC-derived CMs. In particular, qRT-PCR was used to study the expression of relevant early - Brachyury, T-box transcription factor 5 (tbx5), atrial natriuretic factor (anf), nkx2 homeobox 5 (nkx2.5) and beta-myosin heavy chain, (mhc-β) and late -cardiac troponin-I (tnni) - cardiac genes. Interestingly after 14 days, the expression of brachyury, tbx5, β-mhc and ctnni are strongly induced in the printed samples compared to the control bulks, suggesting a higher maturation of the differentiation. Instead, the expression of anf, a gene of the cardiogenic embryonic program, which generally reactivates in case of damage is greater in bulk samples than those printed (Fig. 4).

**Figure 5:**
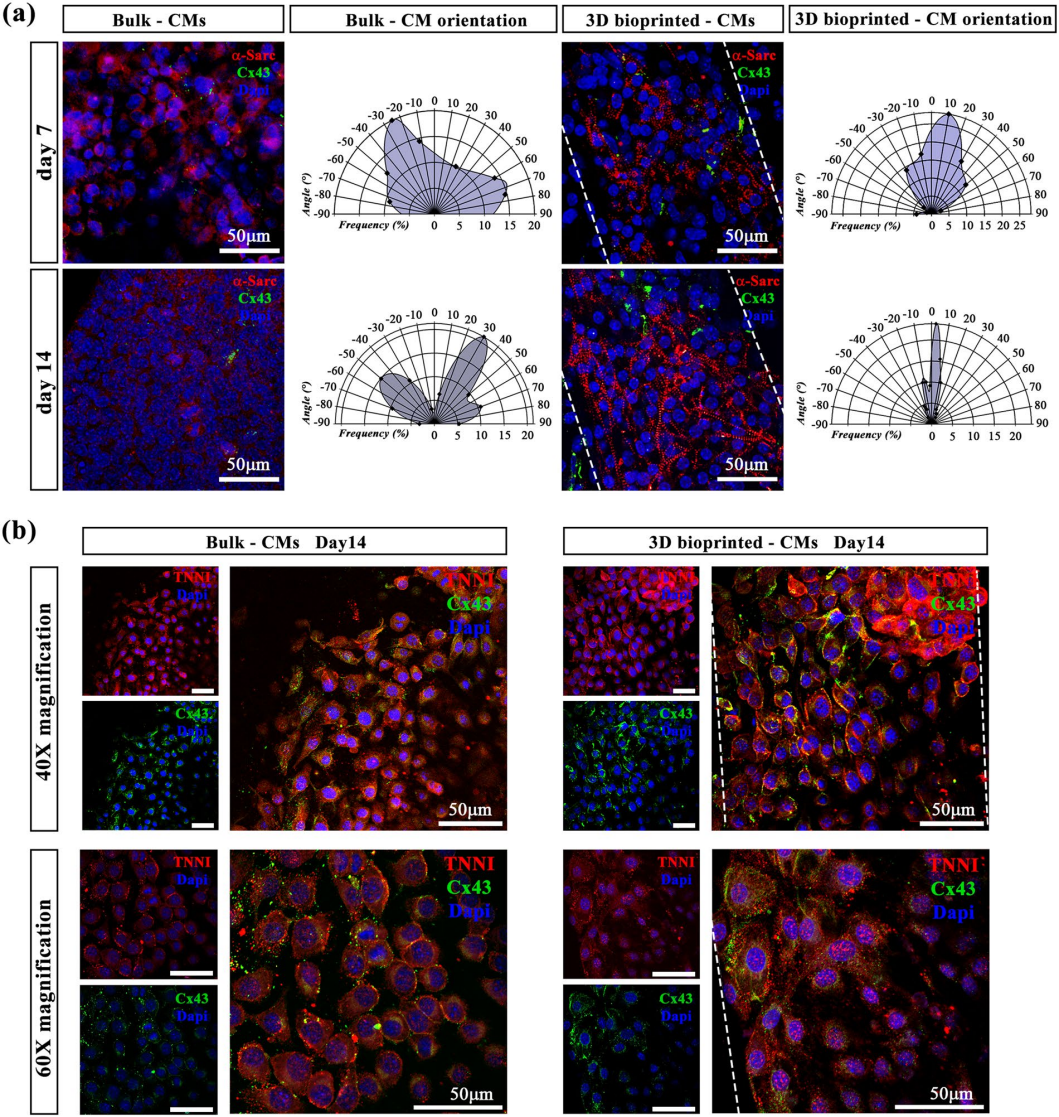
iPSC-derived CM orientation in bulk and in 3D bioprinted scaffolds. (**a**) After 7 and 14 days of culture, CMs were stained against α-SARC (red) and Cx43 (green). Polar charts show the CM orientation in bulk (0° corresponds to X-axis of the image) and 3D bioprinted samples (0° corresponds to the printed fiber direction). N = 5 (**b**) Immunofluorescence assay indicating TNNI (red) and Cx43 (green) expressions after 14 days of culture in Bulks and in 3D bioprinted constructs. Magnification: 40X in upper panels and 60X in lower panels. Scale bars represent 50 μm.

The overexpression of these genes in 3D bioprinted samples might be related to a better and more functional organization of iPSC-derived CMs within the printed oriented hydrogel fibers. For this reason, we performed immunofluorescence stainings labelling cardiac proteins – α-sarcomeric actin (α-SARC), cardiac troponin (TNNI) and connexin 43 (Cx43). The obtained results revealed a significant difference in terms of protein expression and overall cell organization. In fact, iPSC-derived CMs embedded in bulks did not show any myocardium-like organization showing a random cellular orientation. On the contrary, 3D bioprinted iPSC-derived CMs revealed a higher long-range cellular organization into the hydrogel fibers, with a preferential cell orientation along the printing direction (Fig. 5). Such difference in term of cell orientation can be better appreciated in the polar graphs reported in Fig. 5a in which it can be easily noticed that bulk hydrogels are characterized by wide angular distributions (i.e. no organization) while those relative to 3D bioprinted samples are extremely narrower. Moreover, stainings for TNNI and Cx43 in the panel b suggested both cellular maturation and intercellular communication. These data represent a great milestone, confirming that 3D bioprinting of iPSC-derived CMs is an excellent approach to enhance the biofabricated tissue functionality *in vitro* (Supplementary video S2 and S3).

### Multi-cellular cardiac tissue bioprinting

The development of 3D functional heart tissue with specific biological and mechanical properties is one of the greatest challenge in the context of tissue engineering.

Our results demonstrate that, the bioprinting process promotes better differentiation and more functional CM organization, due to cell geometric confinement and spatial orientation within the fibers. Subsequently, in order to promote vascularization support and to recapitulate the intercellular connections present in the cardiac tissue, we intercalated endothelial precursor cells within the 3D cardiac structure.

To this end, we used HUVEC as a model of the endothelium. This cell line – as shown in the paragraph 3.4.2 – further allows to precisely distinguish host’s vessels (potentially infiltrated in the grafted constructs) from those generated in and from the engineered implants. In the multi-cellular bioprinting experiments, HUVEC were resuspended in fresh bioink (containing exclusively 4% w/w ALG and 1% w/w PF) diluted to 6 × 10^6^ cells/mL while the bioink containing iPSC-derived CM was formulated as described in paragraph 3.3. HUVEC and iPSC-derived CM were bioprinted to form three heterogeneous structures with different spatial organization of the two cell types to evaluate potential influences on the formation of a vascular network and on the differentiation and organization of CM. In the first geometry, called Janus, the two different cell lines were precisely compartmentalized within each laid fiber while the other two structures were generated by alternating two layers of HUVEC with two (2:2:2:2:2) or four (4: 2: 4) iPSC-derived CM layers (\6a).

#### *In vitro* characterization of multi-cellular cardiac tissue

**Figure 6:**
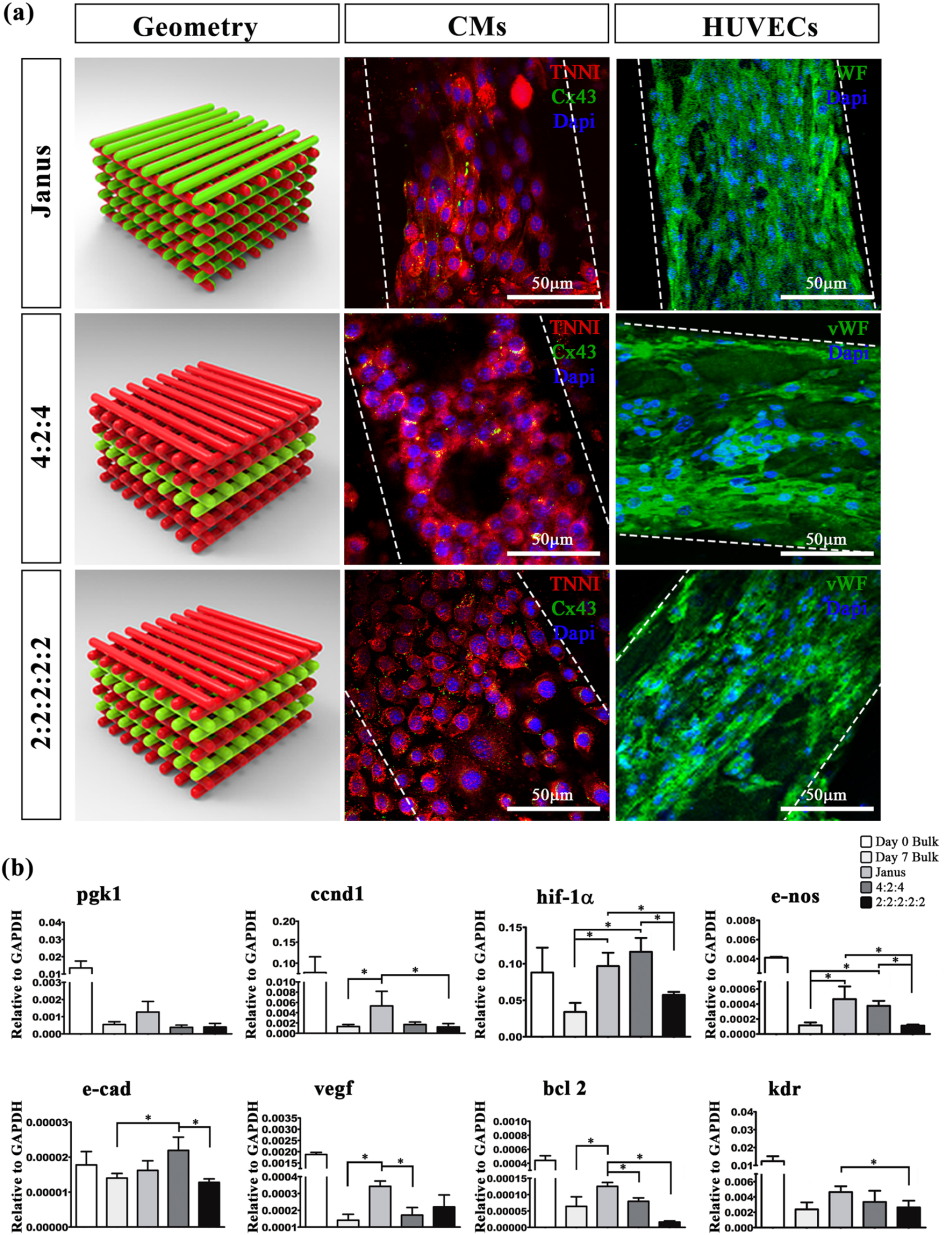
Multi-cellular 3D bioprinted cardiac tissue constructs. (**a**) Representative images showing TNNI (red) and Cx43 (green) expressions in CMs and vWF (green) labelling in HUVEC, after 7 days of culture, printed in three different spatial geometries. Janus constructs contained the two different cell lineages within the each laid fiber; 4:2:4 and 2:2:2:2:2 structures were printed altering two layers of HUVEC with two or four layers of CM. Scale bars represent 50 μm. (**b**) Relative gene expression regarding angiogenesis (hif-1α, e-nos, pgk1, vegf, kdr, e-cad), apoptosis (bcl2) and proliferation (ccnd1). Error bars represent ± SEM. Student’s t test, *p < 0.05; N = 4.

To evaluate the organization of the two cell types within multi-cellular bioprinted samples, we performed immunofluorescence analysis after 7 days of culture. Interestingly, HUVEC developed large endothelial-like structures (i.e. monolayer of cells) with a diameter of about 100 μm independently of their spatial organization. Most notably, scaffold printed with Janus and 4:2:4 geometry promoted greater homogeneity of HUVEC distribution on the fiber surface compared to the 2:2:2:2:2 geometry (Fig. 6a), without presenting decellularized areas on the outer fiber perimeter (confocal z-stack video of Janus fiber is available in supplementary information). Surprisingly, iPSC-derived CM did not form any functional organization in the three multi-cellular bioprinted structures (similar to the one observed in the case of single iPSC-derived CM bioprinting – see paragraph 3.3). This might be explained in terms of too short culturing time and medium formulation for the co-culture of the two cell types: in fact, both of them require specific growth factors that in the employed co-culture medium (CM differentiation medium and EGM2, ratio 1:1) were diluted, thus possibly being inadequate to support CM organization/functionality.

qRT-PCR analysis showed a significant increased expression of genes associated with angiogenesis, including hypoxia-inducible factor 1-alpha (hif-1α), nitric oxide endothelial synthase (e-nos) in Janus and 4:2:4 constructs compared to 2:2:2:2:2 structures and bulk at day7. Furthermore, vascular endothelial growth factor (vegf), vascular endothelial growth factor receptor 2 (kdr), b-cell lymphoma 2 (bcl2) gene expression (related to apoptosis), and cyclin D1 (ccnd1, measuring the proliferation index), were significantly higher in Janus constructs than in the other two types of geometries and control at day7, resulting in improved endothelialisation, prevention from apoptosis and higher rate of cell proliferation. However, the expression of e-cadherin (e-cad) was significantly higher in the 4:2:4 constructs rather than 2:2:2:2:2 and day7 bulk samples (Fig. 6b). In this case, qRT-PCR did not reveal significant differences between the three types of geometry in the expression of important early (brachial, tbx5, nkx2.5 and β-mhc) and late (ctnni) genes (data not shown) because the culture protocol has been developed to promote the correct angiogenic process *in vitro*.

All the results of the gene expression concerning the different geometries were normalized on the basis of the number of individual cell populations printed at the beginning of the experiment. A descriptive image of how the correction factor was obtained is shown in Supplementary Figure 5.

#### In vivo engraftment of multi-cellular cardiac tissue

**Figure 7:**
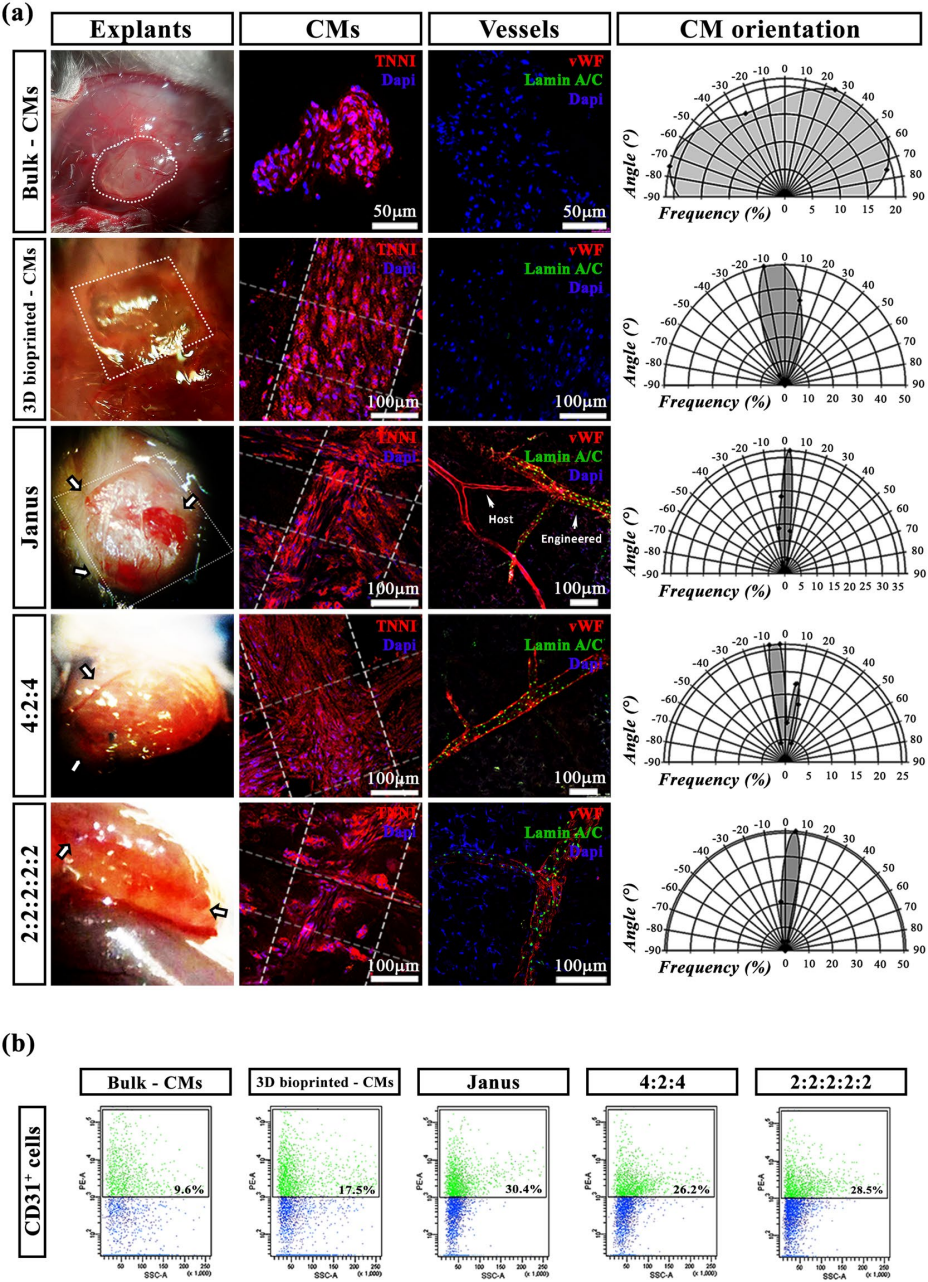
*In vivo* engraftment of 3D bioprinted and bulk hydrogels. (**a**) Implants after 15 days of engraftment, just before explantation (left column). TNN1 immunofluorescence staining used to detect CM and to study their orientation (middle column). The presence of vasculature was identified by vWF labelling (red) while to distinguish between host’s and engineered vasculature we performed Lamin A/C staining that univocally identifies capillaries originated from human endothelial cells (left column). Polar graphs represent the orientation of CM, with 0° corresponding to the fibers deposition direction (printed samples) or the X-axis of the image (bulk sample). Scale bars represent 50 μm and 100 μm. (**b**) Scatter plots indicating the percentage of CD31+ cells after the digestion of subcutaneous transplants with different geometries.

To prove a real advantage of the presented multi-cellular bioprinting approach in assembling more functional constructs for heart tissue engineering/modelling, samples were implanted *in vivo* subcutaneously in NOD-SCID mice. In particular, five different constructs were implanted: i) iPSC-derived CM in bulk hydrogels; ii) iPSC-derived CM in 3D bioprinted constructs; iii) Janus; iv) 4:2:4 and v) 2:2:2:2:2. (i) and ii) were used as controls. All the samples were pre-cultured *in vitro* for 7 days and then grafted *in vivo*. The implants were explanted two weeks later and analysed by immunofluorescence, FACS and qRT-PCR. As shown in Fig. 7, an ameliorated vascularization was found in the cases of multi-cellular bioprinted structures compared to the two controls devoid of endothelial cells (i.e. CMs in bulk and CMs 3D bioprinted). Interestingly, a particular abundant number of blood vessels was found within the implanted Janus constructs. This was further confirmed by immunofluorescence analyses in which it was evidence the formation of branched vascular capillaries and a greater organization and orientation of CMs. Most notably, we observed for Janus constructs well-developed vascular network of human origin (originated by HUVECs and univocally distinguished with human Lamin A/C staining) with the host’s one integrated, reasonably favoured by the paracrine signalling exerted by HUVECs located in the implants. Finally, the orientation of iPSC-derived CMs was also studied for all samples revealing that the 3D bioprinting technique can be used to effectively direct CM alignment with a significant improvement in the overall cell organization (CM orientation – *Janus*: −10° < α < +10°; *4:2:4* and *2:2:2:2:2* −10° < α <+ 20°; CMs 3D bioprinted −30° < α < +30° and CMs in bulk −90° < α < +90°). Interestingly, as shown in the polar graphs, the Janus model exhibits the sharpest distribution (angle interval between −10° and +10°) among the tested geometries, revealing a potential beneficial interaction between HUVECs and CMs in tissue organization when bioprinted with defined organization.

The data achieved were confirmed by flow cytometry (FACS) experiments, the labeling for cluster of differentiation 31 (CD31), a known endothelial factor, showed that the Janus geometry produced the highest number of positive cells compared to the other conditions, confirming the best performance even *in vivo*.

**Figure 8:**
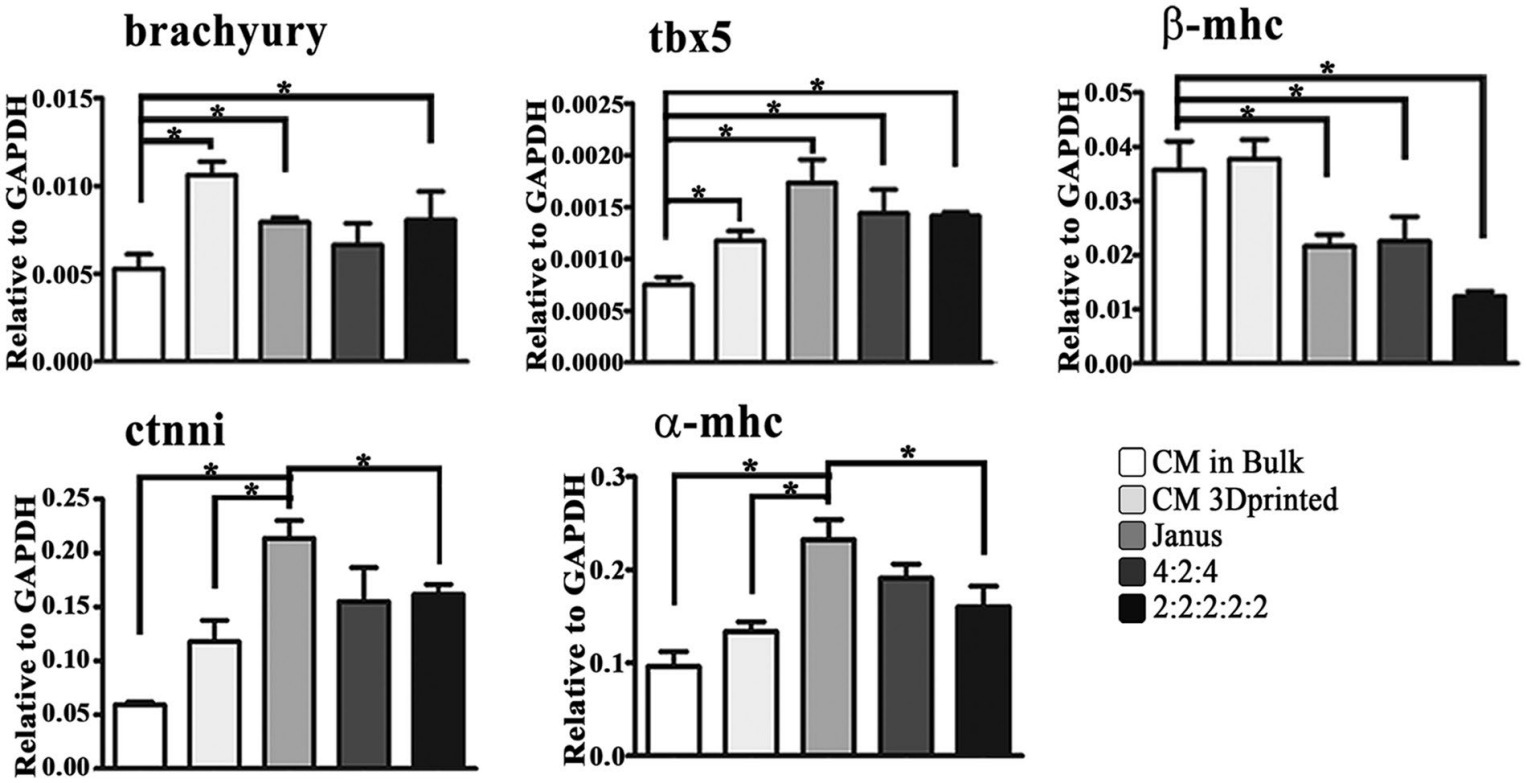
Gene expression *in vivo*. Relative gene expression related to cardiac early genes (brachyury, tbx5, β-mhc) and cardiac late gene (ctnni, α-mhc) in 3D printed multi-cellular constructs compared to CM in bulk structures and 3D bioprinted CMs *in vivo*. Error bars represent ± SEM. Student’s t test, *p < 0.05.; **N** = 3.

On the cardiac side, the construct printed in JANUS geometry produced greater maturation than CMs. This surprising data has been validated by qRT-PCR experiments, which demonstrate how alpha myosin heavy chain (α-mhc) and cardiac troponin-I (ctnni) – late specific cardiac genes – are more expressed in two-faced geometry (Fig. 8).

The obtained results allowed us to state that our multi-cellular bioprinting method guarantees functional infiltration and integration of the host’s vasculature into the bioprinted constructs, providing a rapid blood supply to the implant, avoiding necrosis and favouring the orientation of the heart-like engineered tissue.

## Discussion

The results obtained in this study provide important advances in the field of *in vitro* functional heart tissue generation. Murine iPSCs - likewise embryonic stem cells - are usually grown on a layer of MEF to prevent differentiation. This feature represents an important limitation for obtaining pure cultures of iPSCs. In our previous work, we have shown how iPSCs can be grown - in the absence of feeder layers - in 3D PEG-fibrinogen (PF) bulk hydrogels retaining their pluripotency^28^. iPSC colonies grown on MEF and those grown in PF scaffold did not show morphological differences or dissimilar expression of the stem cell markers Oct4, Sox2 and Nanog. However, cellular distribution in bulk constructs was inhomogeneous due to the migration of iPSC to the outer zone of the hydrogels. This is a typical drawback of bulk hydrogels with cells embedded in the core volume of the sample always suffering the lack of oxygen and nutrients (limited by molecular diffusion).

To overcome this issue, in this work we firstly bioprinted undifferentiated iPSCs within porous structures composed of parallel fibers to eliminate diffusion related problems, obtaining an homogeneous culture biosystem that guarantees an accurate supply of nutrients in all areas of the 3D structure.

Next, we bioprinted prototypes with only iPSC-derived CMs, which showed the ability to produce myocardial-like tissue with a defined cell spatial orientation that partially resemble the functional native one. This was achieved by precisely tuning the matrix composition and stiffness of the final constructs with the addition of 1% w/w PEG-DA monomer within the PF/ALG bioink.

Then, the fully exploit the potentialities of the designed MPH, The core of this system is composed of a microfluidic device bearing a Y-junction (2 inlets, 1 outlet) in which the flows of two different bioink can be precisely and independently controlled by programming external microfluidic pumps. The reduced dimensions of the channels (cross-section 500 × 500 μm) generate an extremely small dead volume (<2 μL) that allows the rapid switching between the two bioinks during printing. This is a key and unique feature of our system which has already shown excellent potential for artificial skeletal muscle generation^29^, that permits to build high-resolution, heterogeneous structures containing iPSC-derived CM and HUVEC that potentially better mimic the native cardiac tissue. This feature offered by our system – i.e. highly precise and repeatable compartmentalization in 3D of multiple cell types – is hardly achievable with other techniques and thus represents a unique advantage of the presented system. In the specific case of heart tissue, the functional contraction of the myocardium is orchestrated by several factors such as the right sequence of electrical stimulation propagation and the defined orientation of the CMs. Thus, in cardiac tissue engineering, obtaining a proper organization of CMs represent a critical goal that, if not achieved, strongly limit the reliability/functionality of the engineered construct. As an example, a culture of CMs without spatial orientation would generate a multidirectional contraction that would produce an overall force tending to zero. On the contrary, in this study we demonstrated that CMs embedded in 3D bioprinted fibers are strongly influence by the surrounding matrix orientation, sensing the direction of fibers. This, in turn, resulted in a better and more functional organization of iPSC-derived CMs that preferentially aligned along fiber printing direction.

Furthermore, it is well known that the maximum nutrient/oxygen diffusion distance for cells to survive without vascularization is ~100–200 μm^30^. Thus, the creation of larger, macroscopic constructs requires the assembly of a vasculature. Several approaches have been developed to promote mass transfer of nutrients and oxygen into engineered tissues, including growth factors that stimulate angiogenesis, but the results are still far from being satisfactory. To this end, we have assembled heterogeneous constructs containing endothelial cells (HUVECs) and iPSC-derived CMs. In this case, the bioprinting strategy consisted of testing 3 different cell spatial organization to determine whether or not there is a significant influence of initial cell spatial organization over the formation and integration of neo-vessel like structures. Such multi-cellular scaffolds were designed so as to have not only a different initial spatial organization of cells, but also different relative volumes occupied by the two cell types. In fact, in the Janus geometry, both cell types occupied 50% of the scaffold volume while in the other two constructs HUVECs occupied only 20% (4:2:4) or 40% (2:2:2:2:2) of the volume. The obtained results demonstrate that Janus cell organization proved to be the most effective with the generation of vessel-like structure with a lumen of about 150 μm. This could be explained in terms of paracrine signalling between the two cell populations that, in Janus constructs, were deposited with the highest cell proximity throughout the scaffold.

In view of *in vivo* experiments, the possibility to have an already pre-formed vasculature represents a great advantage as this could potentially anastomose with host’s vessels and rapidly supply the whole implanted sample. Interestingly, the engineered endothelium never branched randomly during *in vitro* culture, always growing confined to the surface of printed fibers. This feature demonstrates the key role of guidance of printed fibers that should be further exploit in the future to assemble more complex vascular network.

Furthermore, we demonstrated the feasibility of bioprinting multi-cellular constructs that can mature in vascularized functional tissues *in vivo*, indicating potential utility in various translational applications. To the best of our knowledge, this is the first study in which it is shown that bioprinted endothelial cells can effectively develop vasculature in the transplanted tissues, recalling to the same time host’s supporting vessels. These promising results allow us to consider a potential approach for innovative reconstructive therapy directed to the revascularization of ischemic or damaged organs in which the restoration of blood flow could be of great support to counteract cell death and promote regeneration.

## Methods

### Animal procedures

All animal procedures were performed in compliance with the European Convention on Animal Care and approved by the Institutional Animal Care and Use Committee (Italian Ministry of Health). Animals have also received animal care in accordance with the guidelines from Directive 2010/63/EU of the European Parliament on the protection of animals used for scientific purposes.

### iPSC generation

#### Skin fibroblasts isolation

Skin fibroblasts (SFs) were obtained from of 1-day-old C57BL/6 J mice (Charles River Laboratories, Wilmington, MA, USA).

The skin was separated from the rest of the body (n = 10), rinsed with PBS solution (Sigma) and incubated for 45 min at 37 °C in digestion buffer (10 mL for 5 mouse skin) composed of 0.25% trypsin without EDTA (Sigma) and Dispase (BD Bioscience) at 1:1 ratio. The epidermis easily separated from the dermis, which was minced into small pieces and incubated in a shaking water bath for at least 1 hour at 37 °C in a solution of 0.25% Collagenase type I (Worthington Biochemical Corporation). After vigorously pipetting and filtering through 70 μm filter for achieving a single cell suspension, cells were centrifuged at 1200 rpm for 5 min. The cells were resuspended in Dulbecco’s Modified Eagle Medium (DMEM, Gibco, 1×), 20% fetal bovine serum (FBS, Gibco) and 1% Penicillin-Streptomycin (5,000 U/mL), transferred into T75 flask and incubated at 37 °C with 5% CO_2_ for 3 days. SFs were split using trypsin-EDTA and expanded for 2 more passages before undergoing the reprogramming procedure.

#### Fibroblast reprogramming and iPSC generation

Lentiviral vectors employed for the induction of reprogramming via OSK factors harboured bicistronic plasmids expressing oct4 and either sox2 or klf4. The original vector was kindly provided by Prof. L. Naldini (Fondazione San Raffaele, Milan, Italy). To induce reprogramming, SFs were exposed to a mixture of equal volumes of the two OSK lentiviral supernatants in the presence of 4 μg/mL polybrene. On day 7, transduced SF were trypsinized and plated on inactivated mouse iMEF-feeder layer (Millipore, Billerica, MA, USA) at a density of 1.6 × 10^3^ cells/cm^2^. After 24 hours, DMEM medium was replaced with iPSC reprogramming and expansion medium composed of KnockOut DMEM (Invitrogen) containing 20% KnockOut Serum Replacement (Invitrogen), 2mM L-glutamine, 100 μM non-essential amino acids (NEAA), 10 ng/mL bFGF (Peprotech), 500 μM VPA (EMD Biosciences), 100 μM β-mercaptoethanol (Invitrogen), 1000 U/mL leukaemia inhibitory factor (LIF, Millipore), penicillin (100 U/mL), and streptomycin (100 mg/mL). The cells were fed with iPSC expansion medium every day and monitored daily for the formation of colonies, which typically occurred between days 25 to 30 post-infection. Colonies with ES-like morphology were picked and expanded consecutively in 48-, 24-, 12-well plate and in 35 mm tissue culture dishes.

### Evaluation of iPSC pluripotency

Comparative analyses of the obtained iPSC lines were carried out to verify their pluripotency by evaluating the presence of stemness markers (Oct4, Nanog and SSEA1) by means of immunofluorescence (IF) analyses and quantitative RT-PCR (qRT-PCR) (oct4, nanog sox2).

### iPSC-derived cardiomyocyte (CM) differentiation and culture

The iPSC differentiation toward the cardiac phenotype was induced by stimulation with 2.5 ng/mL bone morphogenetic protein 2 (BMP2, Invitrogen) for 12 h and Embryoid Bodies (EB) formation method^27,31^. Hanging-drop cell culture protocol was used to generate EB; briefly, iPSC were detached by adding TrypLE (ThermoFisher) for 3 min at 37 °C, counted and diluted to 20.000 cells/mL in CM differentiation medium composed of DMEM high glucose (Gibco), 15% FBS (Invitrogen), 2mM L-glutamine (Gibco), 100 μM NEAA (Gibco), 100 μM β-mercaptoethanol (Invitrogen), 100 U/mL penicillin, and 100 mg/mL streptomycin (EuroClone). Drops, each containing 500 cells and approximately 25 μl in volume, were spotted on the inverted lid of bacterial dishes (60 droplets/lid) and placed in the incubator at 37 °C, 5%CO_2_. After 3 days, EB were collected, transferred in low attachment culture dishes and cultured in suspension for 3 more days. EB were then aspirated and seeded on 0,1% gelatine-coated plates (roughly 4 EB/cm^2^) in CM differentiation medium at 37 °C and 5% CO_2_. EB began to show areas of spontaneous contraction after two days.

Before of bioprinting experiments, beating cells were first detached using pre-warmed Trypsin-EDTA solution for 5 minutes at 37 °C. The cell agglomerations deposited on the tube bottom were enzymatically dissociated with pre-warmed StemPro Accutase (Gibco) at 37 °C for 5 min. The resulting cell suspension was filtered through 70 µm cell strainer and centrifuged at 1200 rpm for 5 min. The supernatant was removed and cells were resuspended in an appropriate volume of biomaterial to a final concentration of 40 × 10^6^ cells/mL.

### HUVEC culture

Human umbilical vein endothelial cells (HUVECs) were purchased from Invitrogen and grown in EGM2 BulletKit (CC3156, Lonza) medium at 37 °C and 5% CO_2_. Cells were split using trypsin-EDTA and expanded until sufficient quantity was obtained for the bioprinting process. HUVEC were cultured in EBM2 supplemented 10% FBS, VEGF (100 ng/mL) and FGF (100 ng/mL) for two hours before being trypsinized, resuspended in fresh bioink solution and bioprinted.

### Microfluidic printing head (MPH) fabrication

Microfluidic printing head (MPH) was fabricated by micromilling 5 mm thick polycarbonate plates that were sealed together with a hot press at 130 °C for 30 min (pressure = 5 MPa)^32^. MPH was designed to bear a Y-junction (2 inlets, 1 outlet) of microchannels having a square cross-section of 500 × 500 μm. The MPH was fluidically coupled to a coaxial needle systems composed of a 25 G (inner) and 19 G (outer) needles. The inner needle was inserted and glued at the end of the outlet channel, while the outer one presented an independent side inlet and was mounted coaxially respect to the inner one.

### 3D bioprinting experiments

#### Bioink formulation

The bioink used in this work was composed of two biopolymers – namely alginate (ALG) and polyethylene glycol monoacrylate-fibrinogen (PF) – at a concentration of 4% and 1% w/w respectively – dissolved in 25 mM HEPES buffer. Irgacure 2959 was added as photoinitiator in the bioink at a concentration of 0.01% w/w. After biopolymer and photoinitiator dissolution, the bioink was filtered (0.22 μm) to guarantee sterile conditions. Finally, 8 × 10^6^ cells/mL iPSC and 6 × 10^6^ cells/mL HUVEC were resuspended in sterile ink.

iPSC-derived CM were diluted to 40 × 10^6^ cells/mL in a slightly modified bioink containing additional 1% w/w PEG-diacrylate (PEG-DA). Such monomer enhances the mechanical stiffness of PF hydrogels and better exploits the full potential of iPSC-derived CM^28^.

#### 3D bioprinting

Bioinks, containing a single cell type and calcium chloride solution (0.3 M), were loaded in 2.5 mL sterile Hamilton glass syringes and supplied to the MPH and external needle, respectively, of the dispensing co-axial nozzle system via microfluidic pumps (neMESYS low pressure, Cetoni GmbH apparatus). The printing speed and the flow rates of the bioink and crosslinking solution were adjusted to obtain hydrogel fibers of approximately 100 μm in diameter (printing speed = 180 mm/min, Q_bioink_ = 5.1 μl/min, Q_CaCl2_ = 4.6 μl/min). For all experiments, 10 layers-thick constructs were printed with consecutive layers perpendicular to each other (i.e. 0–90° fiber orientation) and 50 μm distance between hydrogel fibers in the X-Y plane. The result of the bioprinting process was a printedscaffold characterized by overall dimensions of 8 × 8 × 1 mm^3^. After 3D bioprinting, all scaffolds were collected using a sterile spatula, placed in a 60 mm dish and UV- crosslinked at low light intensity (365 nm, 4–5 mW/cm^2^) for 5 min.

Finally, to remove alginate from hydrogel strands, printed samples were washed with 25 mM HEPES buffer containing 2 mM EDTA for 2 min and then cultured in the respective cell growth medium.

#### Multi-cellular bioprinting

To fabricate multi-cellular constructs containing iPSC derived-CM and HUVEC, we employed our custom MPH. Microfluidic pumps were programmed to fabricate three types of scaffolds with different spatial concentration and organization of the two cell types. In the first type of constructs, pumps were programmed to deliver simultaneously and at the same flow rate (Q_CM_ = Q_HUVEC_ = 2.55 μl/min) the two cell types: as result, hydrogel fiber with a Janus morphology were laid down. In the other two types of constructs, pumps were programmed to deliver alternately the two bioinks. In one case, four layers of CM were alternated to two layers of HUVEC (4 CM: 2 HUVEC: 4 CM) while in the other, two layers of each cell population were alternated (2 CM: 2 HUVEC: 2 CM: 2 HUVEC: 2 CM). For the sake of clarity, we will refer throughout the text to these multi-cellular constructs as Janus, 4:2:4 and 2:2:2:2:2, respectively.

After bioprinting, samples were first washed with EDTA solution (25 mM HEPES containing 2 mM EDTA), and then cultured in culture medium consisting of CM differentiation medium and EGM2 (1:1) for 7 days. The medium was changed every 2 days. The constructs were then analysed by means of qRT-PCR and immunofluorescence.

### Subcutaneous implantation in mice

NOD-SCID mice were used to validate *in vivo* implantation of five different constructs: (i) Janus, (ii) 4:2:4, (iii) 2:2:2:2:2, (iv) bioprinted iPSC-derived CM and v) iPSC-derived CM bulk. In the case of bulk hydrogels, PF plugs were polymerized *in vitro* within silicon moulds and subsequently placed *in vivo* under mice skin. All samples were pre-cultured *in vitro* in co-culture medium for 7 days and then grafted *in vivo*.

#### Mice preparation

Mice were anesthetized with Zoletil (20 mg/kg, intraperitoneal injection - i.p.) and Rompum (5 mg/kg, i.p.). Successively, the area of the planned incision was carefully shaved with electric razor and sterilized with iodine. Finally, the mouse was placed on the operating table, warmed at 37 °C by underneath heating pad.

#### Graft implant procedure

One longitudinal incision of 1 cm in length was made on dorsal midline. From this incision, about 1 cm deep subcutaneous pocket was blunt dissected for implantation. One scaffold sample was placed per pocket. The incision was then closed with silk 4.0 sutures, using interrupted sutures in a sub-cuticolar fashion, with a buried knot. Mice were housed individually for 2 weeks.

#### Explant procedure

Explants occurred 15 days after implantation. Prior to sample retrieval, mice were sacrificed with cervical dislocation and the samples with surrounding tissue were excised and processed for histological analysis.

### Gene expression analysis

Total RNA was extracted from tissue samples using Trizol (Invitrogen). 1 μg of total RNA was reverse transcribed to cDNA using SuperScript III Kit (Invitrogen) to be amplified by qRT-PCR. Syber Green PCR master mix (Applied Biosystems) and primers specific for markers of staminality, angiongenesis, early and late cardiac commitment were used (see supplementary Table 1 for primer sequences). Each sample was analyzed in triplicate using 7900HT Fast Real-time PCR System equipped with SDS software (Applied Biosystems).

### Immunofluorescence assays

Bulks and 3D bioprinted constructs were washed with 1X PBS for 10 min and fixed in 4% PFA for 2 hours. Hydrogels were then rinsed with PBS for 1 h and permeabilized in 0,3% TRITON X-100 (Sigma) for 2 h at RT to gain access to the intracellular antigens. Thereafter, samples were incubated in blocking solution containing 5% Bovine Serum Albumin (BSA, Sigma) and 0.1% Triton X for 1 h to saturate the non-specific sites. The constructs were incubated with primary antibodies diluted 1:100 in 0,5% BSA solution overnight at 4 °C. The primary antibodies were the following: mouse monoclonal antibody against Oct3/4 (BD Transduction Laboratories), Ki67 (Abcam), α-sarcomeric actin (ThermoFisher), Cx43 (Cell signaling technology), rabbit monoclonal antibody against anti-cardiac troponin I (TNNI, Chemicon), sheep monoclonal antibody against Von Willebrand Factor (Abcam). Secondary antibodies FITC or TRITC conjugated (Jackson Immunoresearch) and diluted 1:200 were used. Nuclei were counterstained with DAPI. Laser scanning confocal microscopy (Leica Microsystem) was employed to image the labelled constructs. Analyses were performed in sequential scanning mode to rule out cross bleeding between channels.

The excised implants were fixed in 10% formalin, washed with PBS, incubated in 5% BSA for 3 h at RT and stained overnight at 4 °C by the following primary antibodies diluted 1:100 in 0,5% BSA: rabbit anti-cardiac troponin I (TNNI; Chemicon), sheep anti-von Willebrand (vWF; Abcam), mouse anti-Lamin A/C (SantaCruz Biotechnology). After rinsing, the explants were incubated for 6 h at RT in fluorescent-conjugated secondary antibodies diluted 1:500. Negative controls were tested by secondary antibody incubation alone. All secondary antibodies were negative for non-specific staining. After washing, the slides were mounted with vectashield (ThermoFisher Scientific) plus DAPI.

### Live/Dead assay

The viability of bioprinted iPSCs was evaluated by LIVE/DEAD™ Viability/Cytotoxicity Kit (Invitrogen) at day/s 0, 7 and 14. Two μL of ethidium homodimer and 0.5 μL of calcein AM were diluted in 998 μL PBS, then 200 μL were administred to bulks and 3D bioprinted constructs, and incubated for 45 min in 5% CO_2_ at 37 °C. Living and death cells were observed by laser scanning confocal microscopy (Leica Microsystem). Living cells were detected by calcein AM (green fluorescence), and death cells by EthD-1 (red fluorescence). The number of viable cells was quantified using ImageJ software. The number of cells was calculated by the ratio among the area of each cluster with the area of a known single cell. Then, the viability rate was obtained by comparing the number of viable cells with total number of viable and non-viable cells.

### Evaluation of the cardiomyocyte orientation

Z-stack images were processed using ImageJ software. In particular, the orientation of the CM sarcomeres was calculated as the weighted average of the angles formed by each cell polyline segment with the direction of fiber deposition during bioprinting process (0°). In the case of non-bioprinted samples (i.e the bulk hydrogels), X-axis has been considered as a reference (0°). Angular percentage frequencies were then used to draw polar charts.

### Flow cytometry analysis (FACS) of explants

FACS Canto Cell Analyzer (Becton Dickinson) was used for the quantification of endothelial cells derived from multicellular constructs after explantation. To this end, the tissue was taken from the host, and washed in sterile PBS to remove blood residue. Then the explants were minced in small pieces and incubated in a shaking water bath for at least 90 min at 37 °C in a digesting solution consisting in Collagenase A 2 mg/mL (Roche 10103586), Dispase II 3 mg/mL (Roche, 04942078001) and DNAse I 10 μg/mL (Roche, 1284932). After digestion, the cells were washed in PBS, fixed in 4% formalin and stained with anti - cluster of differentiation 31 (anti-CD31; eBioscience, 12-0311-82) antibody. The labelled cells were detected FACS.

### Statistical analysis

Statistical analysis was carried out using GraphPad (Software Inc., La Jolla, CA, USA). Values presented are mean ± SEM. Differences between sample means at each time point were evaluated with Student’s t-test. P-value of < 0.05 was considered statistically significant. P values for each experiment are shown in supplementary information.

## Electronic supplementary material

Supplementary Information

VideoS1

VideoS2

VideoS3
