# Supplementary material for: A multi-cellular 3D bioprinting approach for vascularized heart tissue engineering based on HUVECs and iPSC-derived cardiomyocytes
Source: Sci Rep. 2018 Sep 10;8:13532. doi: 10.1038/s41598-018-31848-x (PMC6131510; doi:10.1038/s41598-018-31848-x)
Supplement: Supplementary file 1 — Supplementary Information [file 41598_2018_31848_MOESM1_ESM.pdf]

A multi-cellular 3D bioprinting approach for vascularized heart tissue engineering based on HUVECs and iPSC-derived cardiomyocytes.

Fabio Maiullari[1,2], Marco Costantini[3,4], Marika Milan[2], Valentina Pace[2], Maila Chirivì[2], Silvia Maiullari[2], Alberto Rainer[4], Denisa Baci[5], Hany El-Sayed Marei[6], Dror Seliktar[7], Cesare Gargioli[8], Claudia Bearzi[2*], Roberto Rizzi[1,2,*].

[1]Operational Research Unit, Fondazione di Ricerca e Cura Giovanni Paolo II, Largo Gemelli, Campobasso 86100, Italy.
[2]Institute of Cell Biology and Neurobiology (IBCN), National Research Council of Italy (CNR), Monterotondo Scalo, Rome 00015, Italy.
[3]Institute of Physical Chemistry, Polish Academy of Sciences, Warsaw 01224, Poland.
[4]Tissue Engineering Lab, Università Campus Bio-Medico di Roma, Rome 00128, Italy.
[5]IRCCS MultiMedica, Scientific and Technology Pole, Milan 20100, Italy.
[6]Biomedical Research Center (BRC), Qatar University, Doha 2713, Qatar.
[7]Department of Biomedical Engineering, Technion Institute, Haifa 32000, Israel.
[8]Department of Biology, Tor Vergata Rome University, Rome 00133, Italy.

Corresponding Author:
Roberto Rizzi,
Institute of Cell Biology and Neurobiology (IBCN), National Research Council of Italy (CNR)
Via E. Ramarini, 32 00015 Monterotondo Scalo, Rome, Italy
Tel. +39 06 50170 3033
email: roberto.rizzi@cnr.it

Co-corresponding Author:
Claudia Bearzi
Institute of Cell Biology and Neurobiology (IBCN), National Research Council of Italy (CNR)
Via E. Ramarini, 32 00015 Monterotondo Scalo, Rome, Italy
Tel. +39 06 50170 3180
email: claudia.bearzi@cnr.it

Supplementary Information

Supplementary figures:

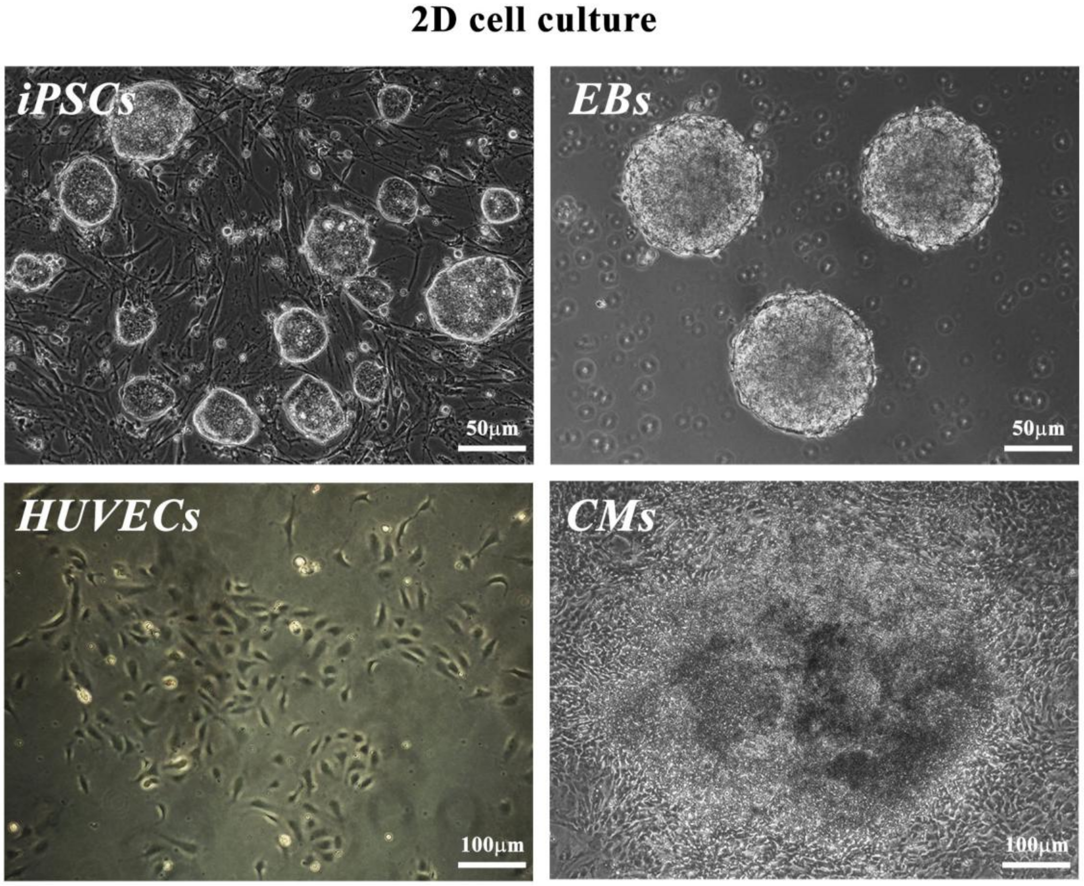

Figure S1. 2D Cells culture. Representative images of iPSCs, EBs, HUVECs and CMs grown in 2D culture in standard condition. Scale bars represent 50μm and 100μm.

(a)

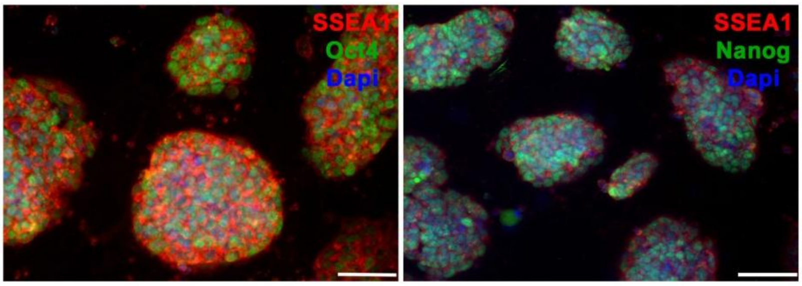

(b)

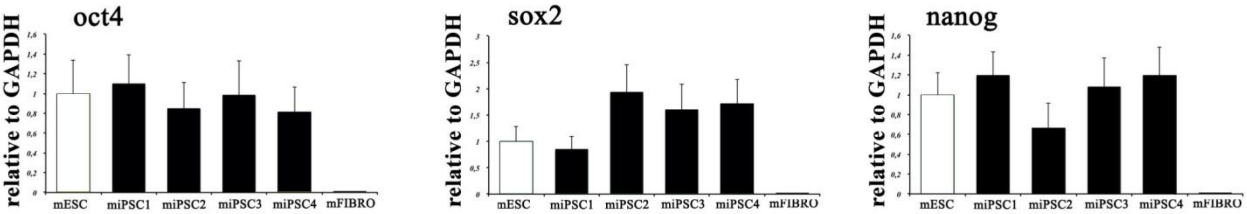

Figure S2. iPSCs pluripotency. (a) Immunofluorescence for stemness markers SSEA1 (red) and Oct4 (green) (left panel), Nanog (red) (right panel). Scale bar represent 200μm. **(b)** qRT-PCR analysis for stemness genes, such as Oct4, Sox2 and Nanog, in obtained murine iPSC compared to murine ESC and murine fibroblasts. Error bars represent mean±SEM. N = 4. Student’s t test did not evidence significant differences in gene expression among the different tested conditions.

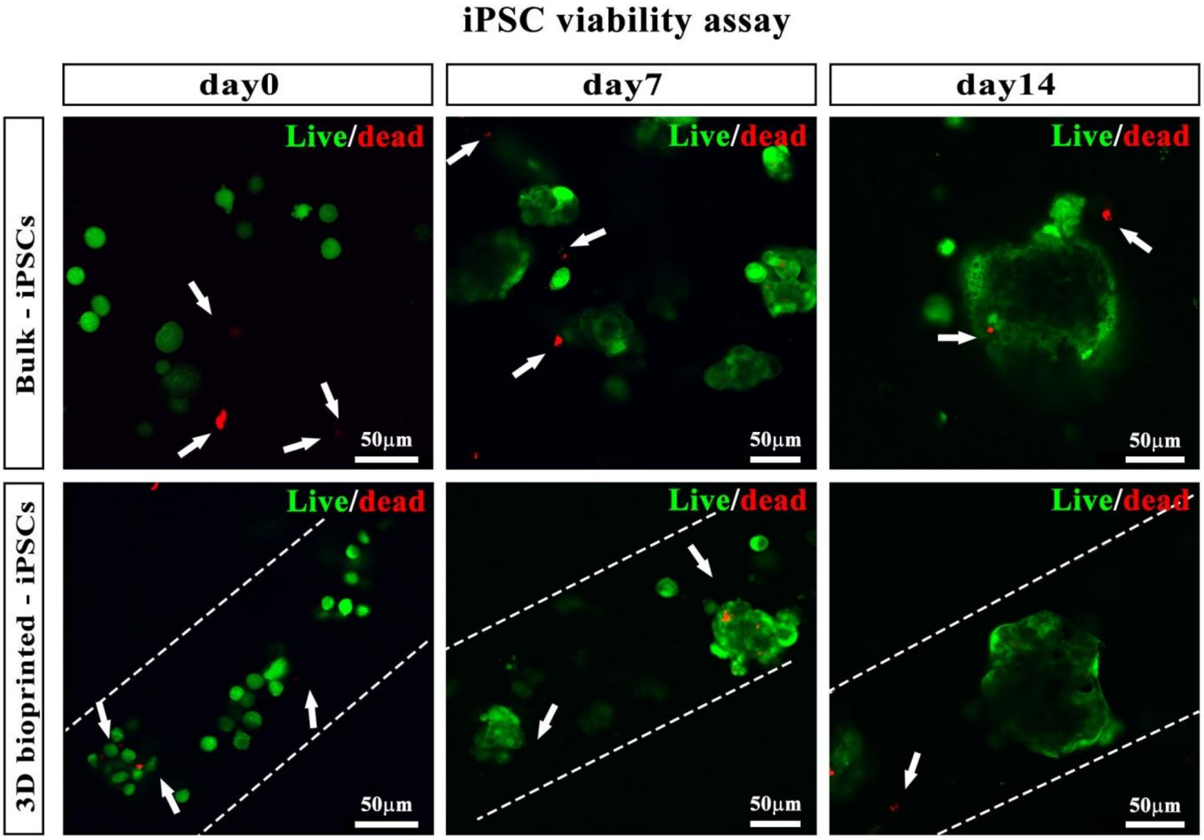

Figure S3. Viability assay of iPSCs. Representative images of iPSC live/dead assay grown in Bulk and 3D bioprinted construct at Day 0, Day 7 and Day 14. Live cells were detected by calcein AM (green), and dead cells by EthD-1 (Red). Scale bars represent 50 µm.

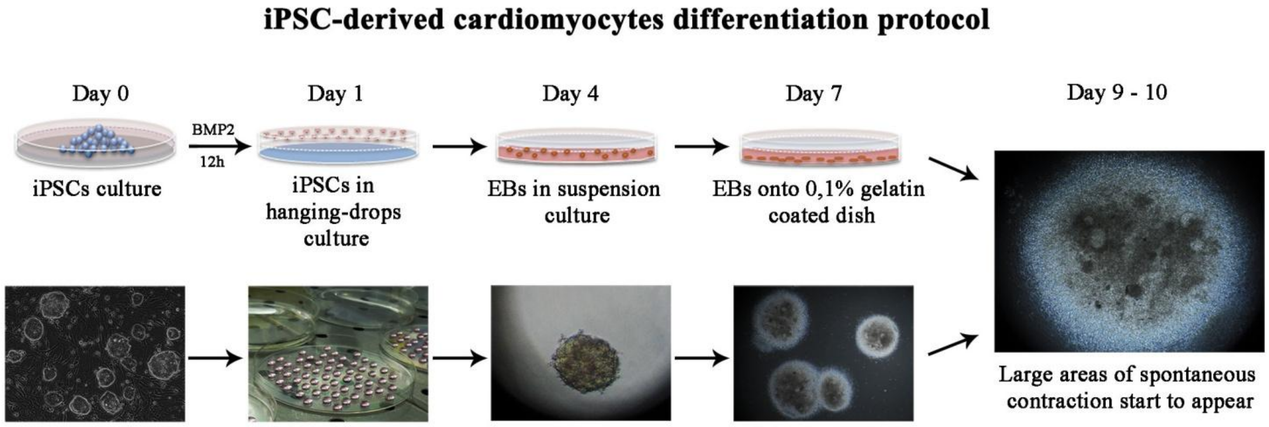

Figure S4. iPSC-derived cardiomyocytes differentiation protocol.

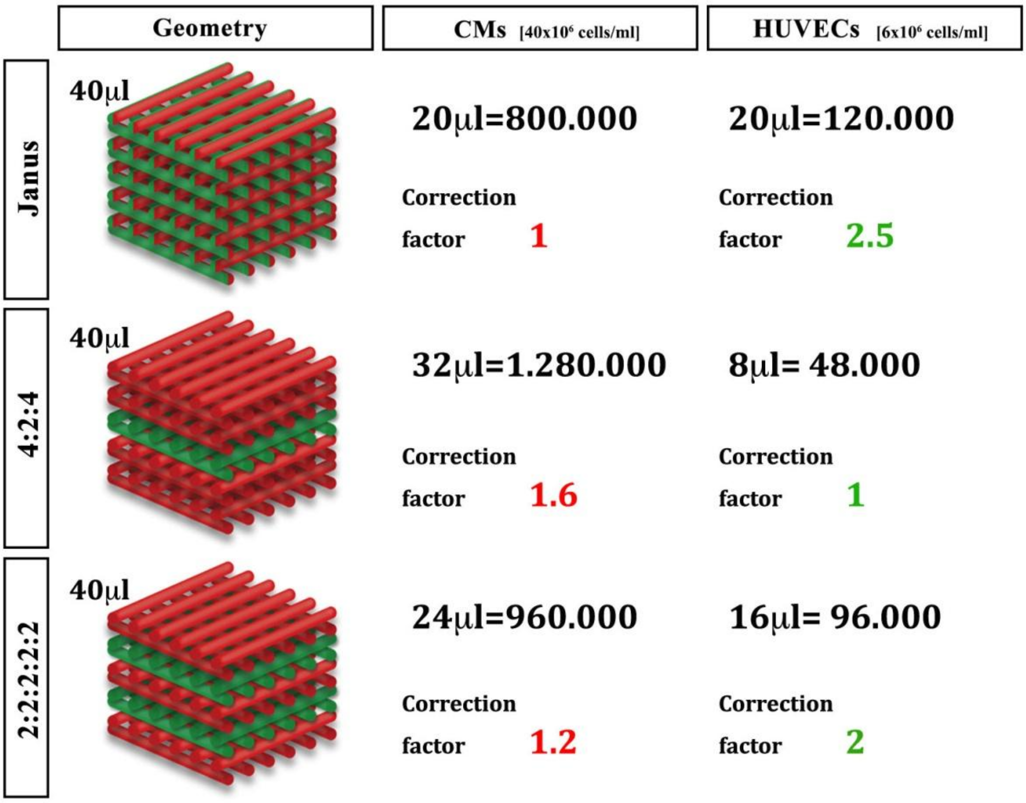

Figure S5. Descriptive image to assess the normalization index .

Table S1. Mouse and human primer sequences for quantitative RT-PCR analysis.

Gene	qRT-PCR primer set	
m_oct4	*forward*<br>*reverse*	CCCTCTGTTCCCGTCACTG<br>ACCTCCCTTGCCTTGGCT
m_sox2	*forward*<br>*reverse*	TGCTGCCTCTTTAAGACTAGGAC<br>CCTGGGGCTCAAACTTCTCT
m_nanog	*forward*<br>*reverse*	CAGGTGTTTGAGGGTAGCTC<br>*CGGTTCATCATGGTACAGTC*
m_brachyury	*forward*<br>*reverse*	CAGCCCACCTACTGGCTCTA<br>GAGCCTGGGGTGATGGTA
m_nkx2.5	*forward*<br>*reverse*	CAAGTGCTCTCCTGCTTTCC<br>GGCTTTGTCCAGCTCCACT
m_tbx5	*forward*<br>*reverse*	CGAAGTGGGCACAGAGATG<br>CACCTTCACTTTGTAACTAGGAAACA
m_anf	*forward*<br>*reverse*	ACAGACCCTGGACAGACACC<br>TGATCCACTGGACAAGGAAA
m_β-mhc	*forward*<br>*reverse*	CGCATCAAGGAGCTCACC<br>CTGCAGCCGCAGTAGGTT
m_tnni	*forward*<br>*reverse*	GCAGGTGAAGAAGGAGGACA<br>CGATATTCTTGCGCCAGTC
m_α-mhc	*forward*<br>*reverse*	*CCAACACCAACCTGTCCAAG*<br>*CTCGTCGTGCATCTTCTTGG*
h_bcl2	*forward*<br>*reverse*	*GCCCTGTGGATGACTGAGTA*<br>*GAAATCAAACAGAGGCCGCA*
h_e-cadherin	*forward*<br>*reverse*	*ACAACAAGCCCGAATTCACC*<br>*GGTGTTCACATCATCGTCCG*
h_hif-1 α	*forward*<br>*reverse*	*ATTTTGGCAGCAACGACACA*<br>*GGGTGAGGGGAGCATTACAT*
h_vegf	*forward*<br>*reverse*	*TCTACCTCCACCATGCCAAG*<br>*TGATGATTCTGCCCTCCTCC*
h_kdr	*forward*<br>*reverse*	*CCCAGGCTCAGCATACAAAA*<br>*CCTCTGTCCCCTGCAAGTAA*
h_pgk1	*forward*<br>*reverse*	*TCTCATGGATGAGGTGGTGA*<br>*CAGTGCTCACATGGCTGACT*
h_enos	*forward*<br>*reverse*	*TCTGCATGGACCTGGATACC*<br>*CGATGGTGACTTTGGCTAGC*
h_ccnd1	*forward*<br>*reverse*	*GATCAAGTGTGACCCGGACT*<br>*TCCTCCTCTTCCTCCTCCTC*
gapdh	*forward*<br>*reverse*	GGCAAATTCAACGGCACA<br>GTTAGTGGGGTCTCGCTCTG

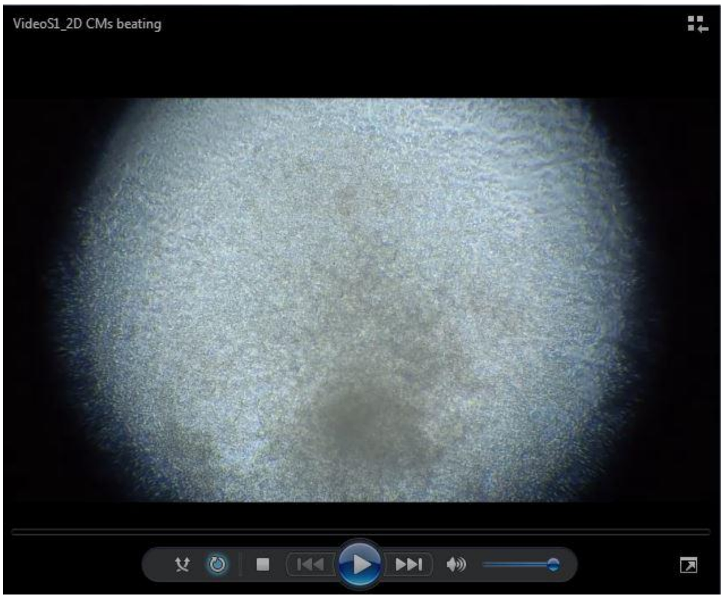

Video S1. CMs self-contraction in 2D culture.

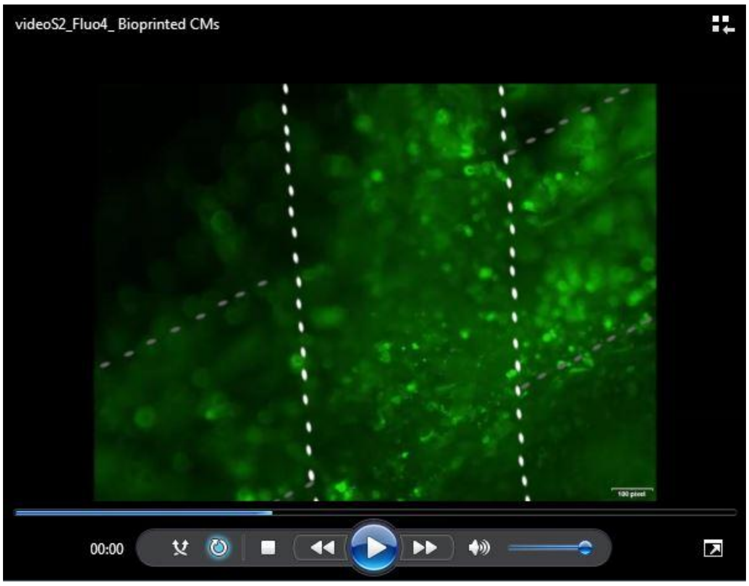

Video S2. CMs contraction in 3DBioprinted construct.

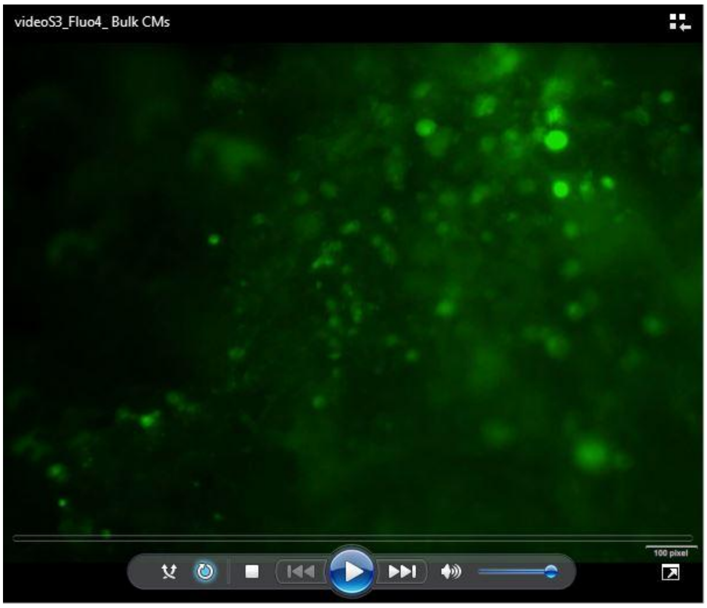

Video S3. CMs contraction in bulk construct.

Supplementary methods:

Ca2+ response in CMs.

The Ca^{2+} -sensitive fluorescent dye Fluo4-AM was administered to the structures to monitor contraction capability of iPSC-derived CMs. At day 14, bulks and bioprinted constructs culture media were removed and the structures were loaded with Fluo4-AM 10μM for 1h at 37°C in Tyrode's solution (NaCl 140mM, KCl 5mM, HEPES 5mM, NaH_2PO_4 1mM, $MgCl_2$ 1mM, $CaCl_2$ 1,8 mM, Glucose 10mM adjusted at pH 7.4). After dye loading, the samples were washed and incubated in fresh Tyrode's solution for 20min, to allow the de-esterification of the intracellular indicator. Cardiac specific stimulation was performed adding caffeine 10mM. Experiments were performed at 25±2 °C and videos of contraction were acquired at 10X magnification (Olympus, AX70).
